# Supplementary material for: Predicting hedgehog mortality risks on British roads using habitat suitability modelling
Source: PeerJ. 2020 Jan 21;7:e8154. doi: 10.7717/peerj.8154 (PMC6979406; doi:10.7717/peerj.8154)
Supplement: Supplemental Information 2 [file peerj-08-8154-s002.docx]

# Supplementary information

Supplementary 1A: Information on the available hedgehog roadkill records used in this study. The information presented is grouped into data providers. Collection methods include: ONR – Online Recording, EAR – Effort Associated Sightings, SPA – Smart Phone App, OPS – Opportunist Sightings, EML – Email recording

| Data provider | Start date | End date | Projects included | Collection methods |
| --- | --- | --- | --- | --- |
| People Trust for Endangered Species (PTES) | 2011 | 2018 | Big Hedgehog Map, Mammals on Roads, Suffolk Wildlife Trust | OLR, EAR, SPA, OPS |
| Mammal Society (MS) | 2000 | 2017 | National Atlas Project, Mammal Mapper, Mammal Tracker | SPA, EAR, OPS, EML |
| Project Splatter (PS) | 1959 | 2018 | Project Splatter | SPA, OPS, OLR, EML |

Supplementary 1B: Raw data of hedgehog roadkill records collected from different citizen science projects


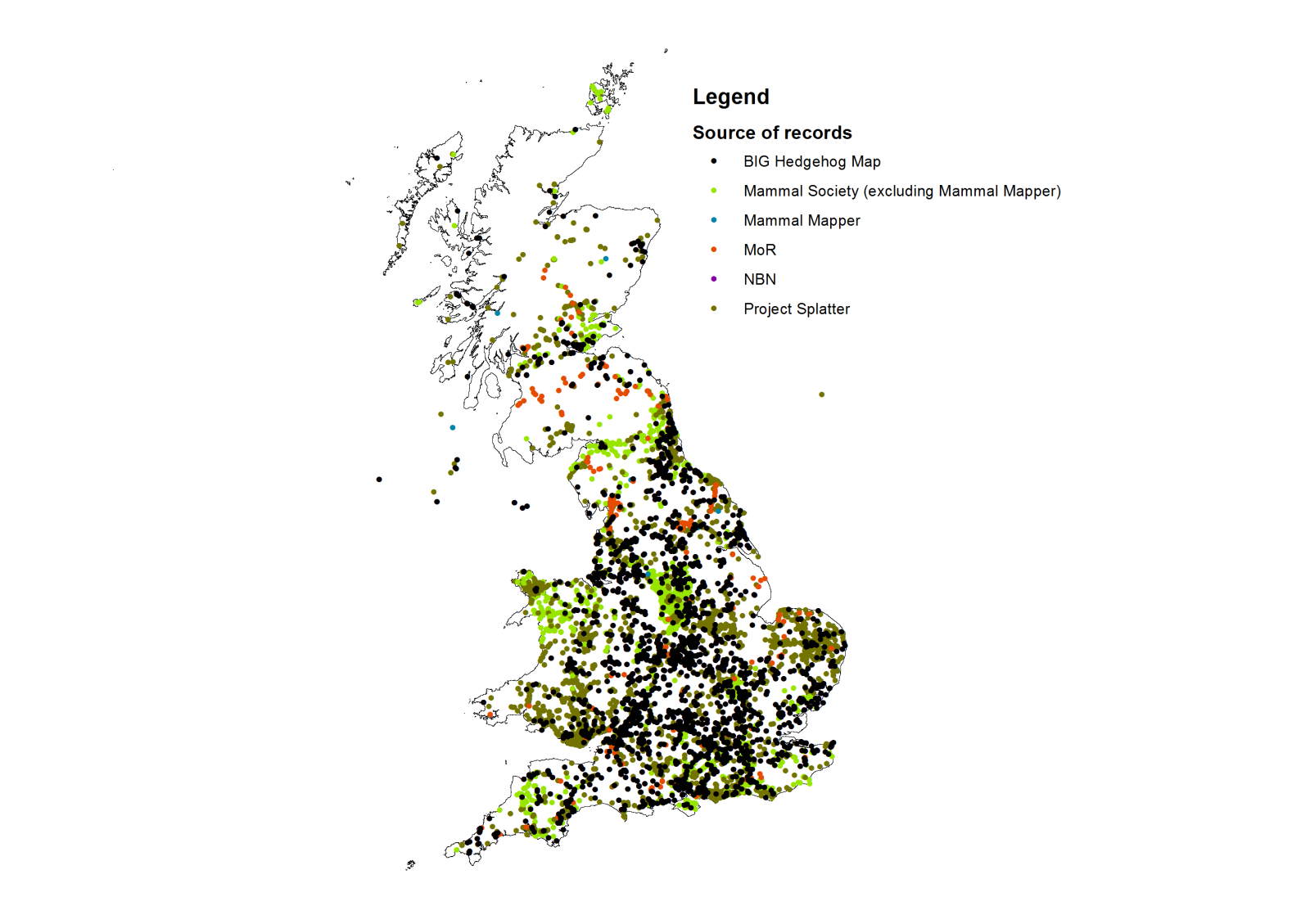


Supplementary 2: Additional information on the preparation of landscape variables for MaxEnt.

A suit of environmental variables were sourced from various datasets with the initial selection being based on the *a priori* expectation that they have an influence on the presence or impact of hedgehogs. The countrywide modelling framework was conducted at a 1-3 km resolution and focused primarily on habitat, road density and climatic variables. The percentage of habitat was calculated using the 2007 Land Cover Map (LCM: Morton et al., 2011) from the Centre for Ecology and Hydrology (CEH). The percentage area of each broad habitat classification was calculated for each 1 km^2^ British National grid square. At 3 km resolution, the habitat percentages were amalgamated from the 1 km resolution grid. The full list of habitats is available in (Figure 1 and Supplementary 5). Road densities were calculated using ArcGIS (ESRI, 2011) by measuring the km of road from the OS road shapefile (<https://www.ordnancesurvey.co.uk/opendatadownload/products.html>) within a 1km grid. Road type specific analyses were calculated by dividing the shapefile into road classifications (minor roads, B roads, Major roads and all roads). The climatic data was sourced from WorldClim (<https://www.worldclim.org/>).

Supplementary 3: Road type classifications and distributions used in this project. Below are the road classifications for minor, B, Major and All road shapefiles used in the road-level models. These shapefiles are based on the OS Open Roads data from the Ordinance Survey (<https://www.ordnancesurvey.co.uk/opendatadownload/products.html#OPROAD>) and divided into types based on the class and function fields.


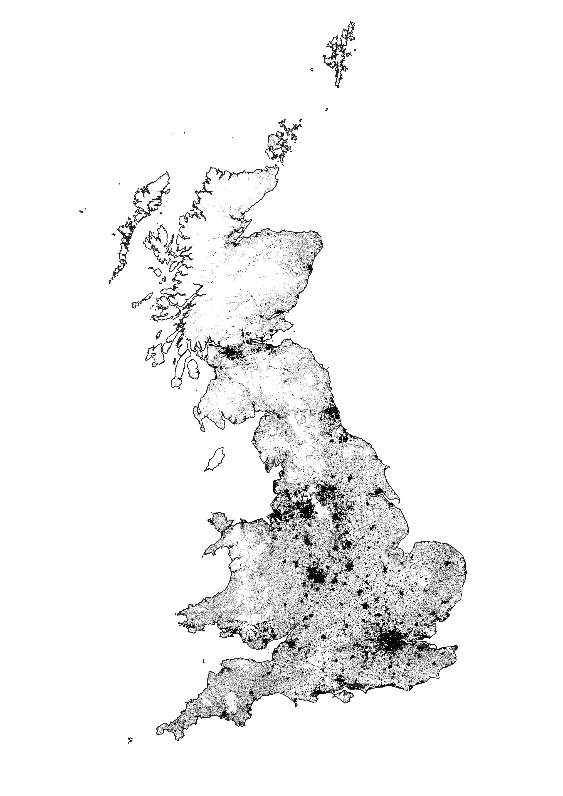

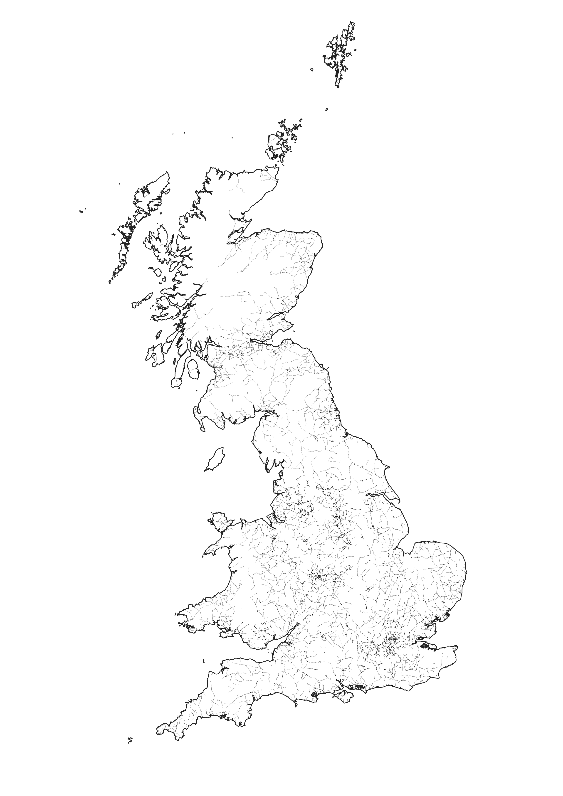

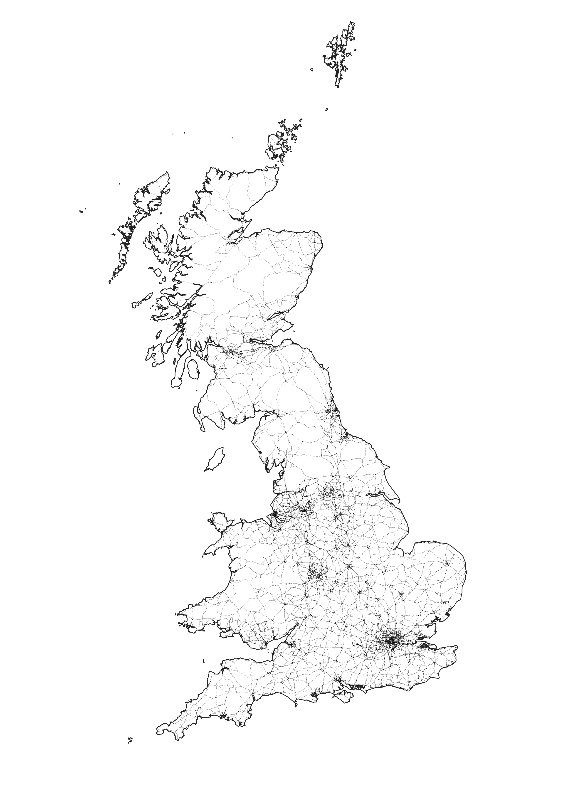

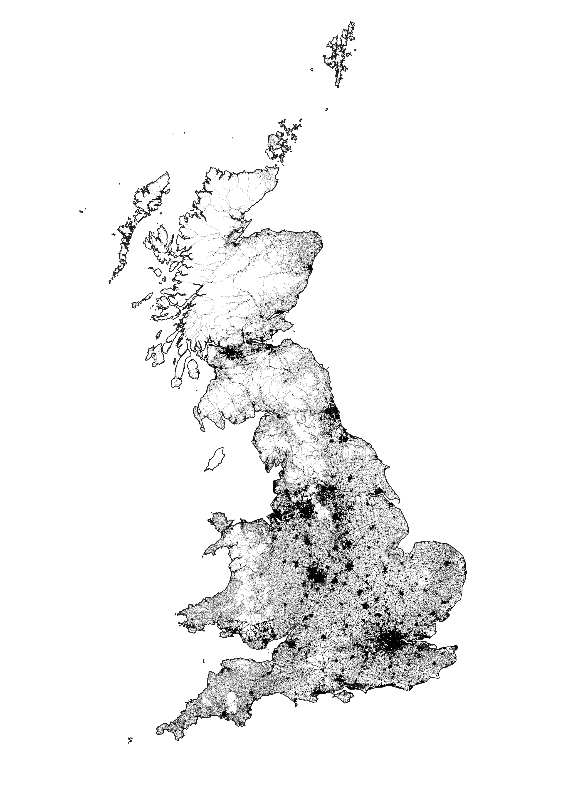


Major roads

All roads

B roads

Minor roads

Supplementary 4: Table representing the model performance of the final national scale (1 km) roadkill HSM using roadkill background data and the road HSM’s (100 m) for models using random background data and roadkill background data. Schoener’s D index scores represent the niche overlap between models using random and roadkill background data

| Background data | Model type | Feature types | Regularisation multiplier | AICc | Full AUC |
| --- | --- | --- | --- | --- | --- |
| *Rdkill* | National | LQ | 1 | 138,246 | 0.72 |
|  | National + Major road | LQ | 1 | 22,054 | 0.50 |
|  | National + B road | LQH | 4 | 10,186 | 0.60 |
|  | National + Minor road | LQH | 4 | 44,442 | 0.71 |
|  | National + all roads | LQHPT | 4 | 79,724 | 0.74 |
| *Rdm* | National | LQHPT | 1.5 | 135,071 | 0.75 |
|  | National+ Major road | LQH | 4 | 22,353 | 0.65 |
|  | National + B road | LQHPT | 4 | 9,593 | 0.60 |
|  | National + Minor road | LQH | 4 | 43,025 | 0.65 |
|  | National + all roads | LQ | 1 | 79,379 | 0.61 |


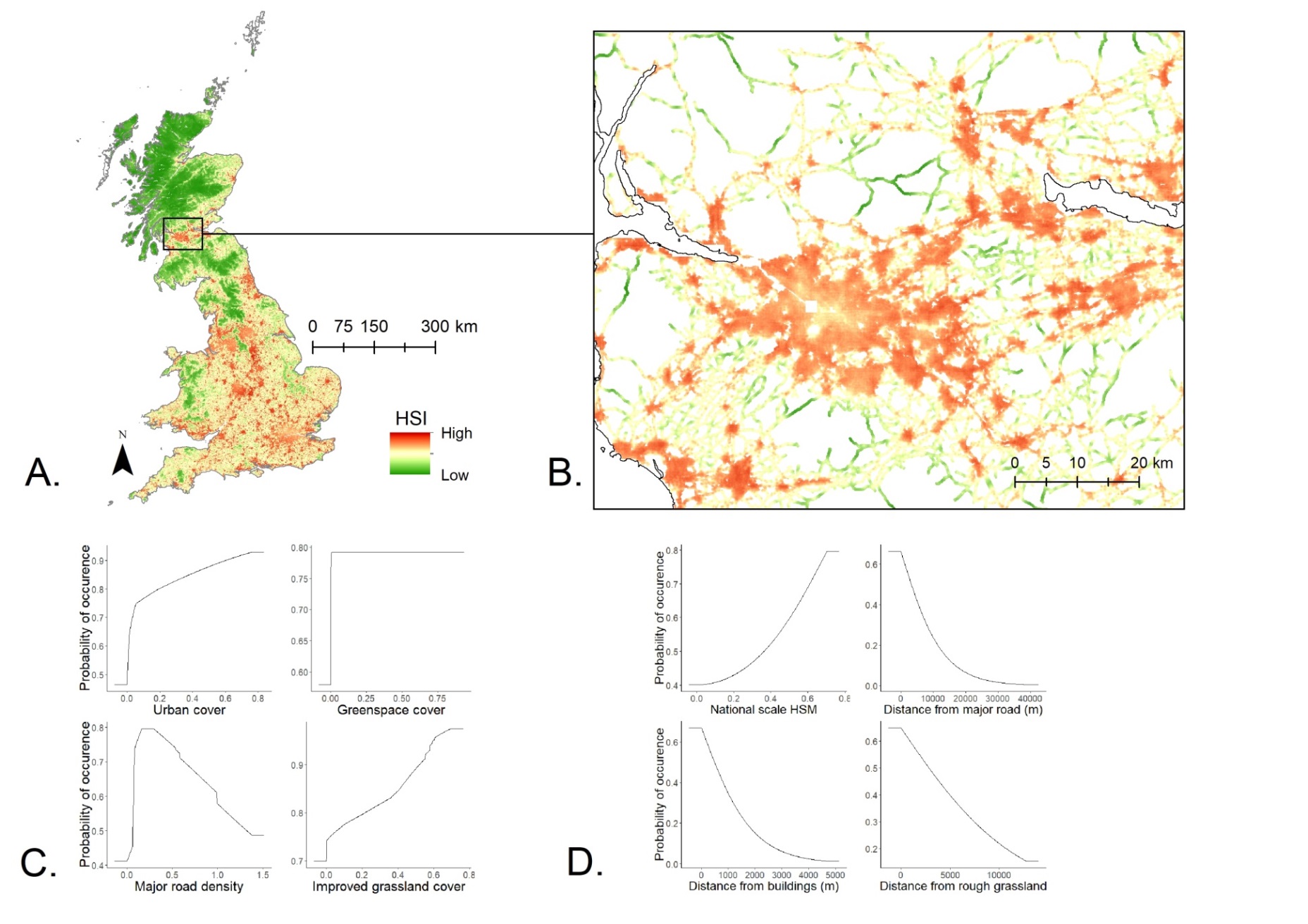


Supplementary 5: Mapped logistic habitat suitability indices (HSI) for *E. europaeus* roadkill at a national scale (A), then at a road level using all road data (B) according to the hierarchical, multi scale model using random background data. The response curves with the highest permutation importance are represented for the national scale (C) and the model using all road data (D).

Supplementary 6: Variable full names of raster files used for Maxent

| **Raster name** | **Variable full name** |
| --- | --- |
| alltraff | All roads traffic |
| arable | Arable cover |
| broad_dens | B road densiy |
| broad | Broadleaved cover |
| conif | Coniferous woodland cover |
| disarable | Distance from arable |
| distbrd | Distance from B roads |
| eucdistbroa2 | Distance from broadleaved woodland |
| dis_build | Distance from buildings |
| eucdistconi2 | Distance from coniferous woodland |
| disfreshw | Distance from freshwater |
| disimpgra | Distance from improved grassland |
| dist_minor | Distance from Minor road |
| dist_mw | Distance from motorway |
| disrougra | Distance from rough grassland |
| dissuburb | Distance from suburban area |
| disurban | Distance from urban area |
| freshw | Freshwater cover |
| Greencover | Green space cover |
| HedgerowDensity | Hedgerow density |
| imp_grass | Improved grassland |
| mw_dens | Major road density |
| minorrd_tr | Minor road density |
| popdens | Population density |
| slope | slope |
| urban | urban cover |


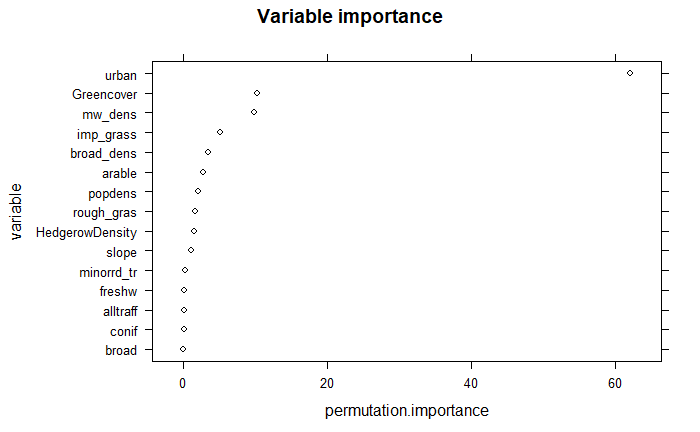


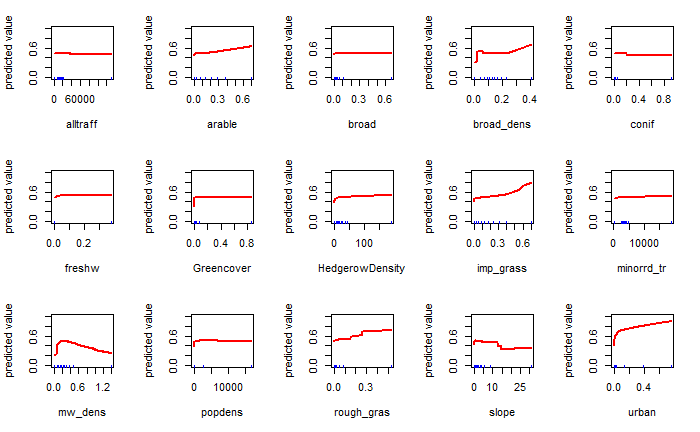


Supplementary 7: Summary of variable importance and response curves of the national model (random background data)


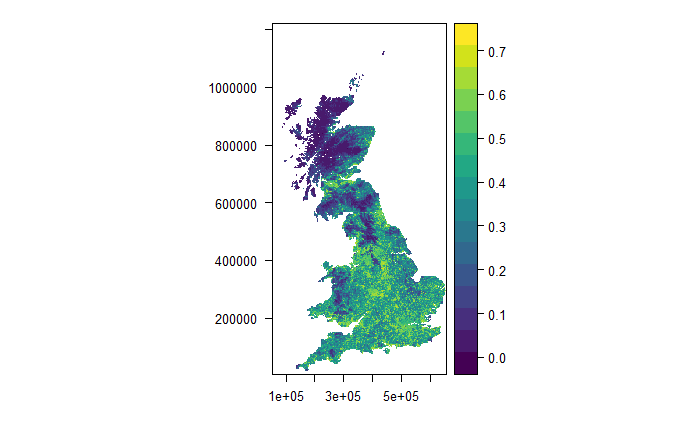


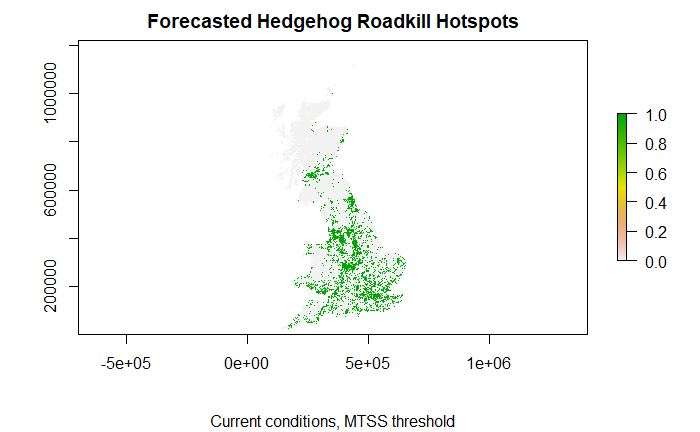


Supplementary 8: Mapped logistic habitat suitability indices (HSI) for hedgehog roadkill and forecasted high-risk of collision areas of the national model (random background data)


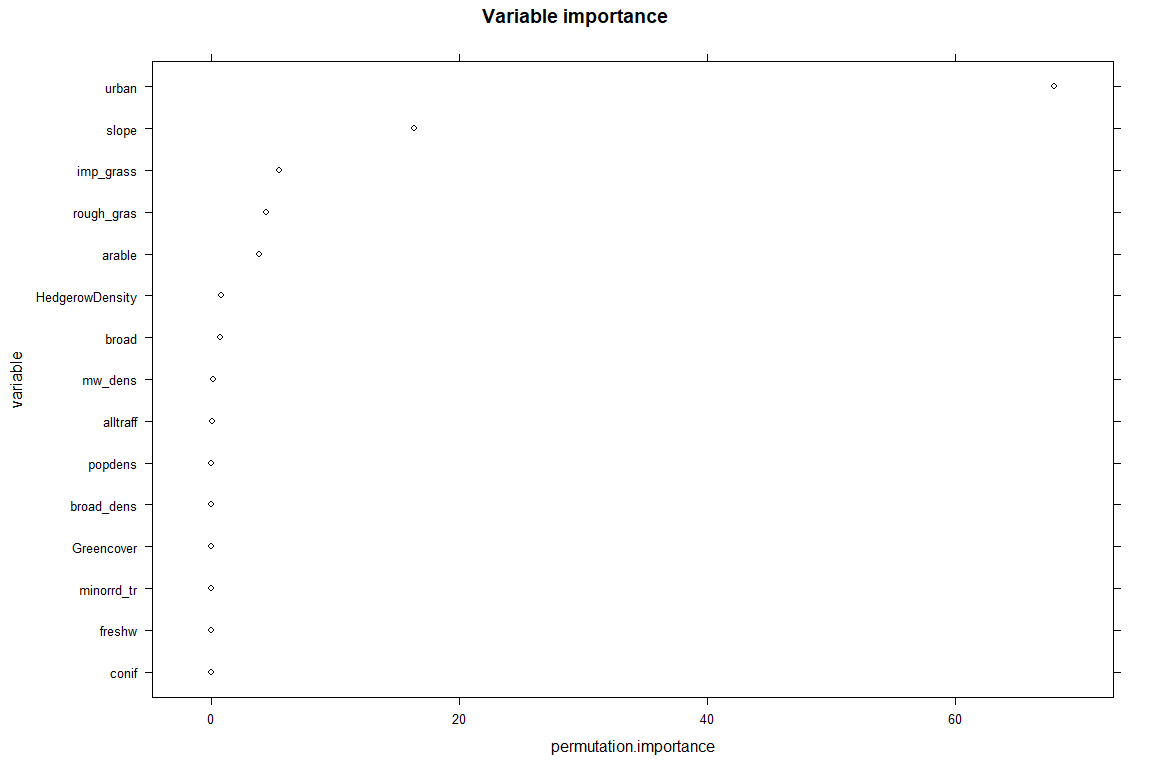


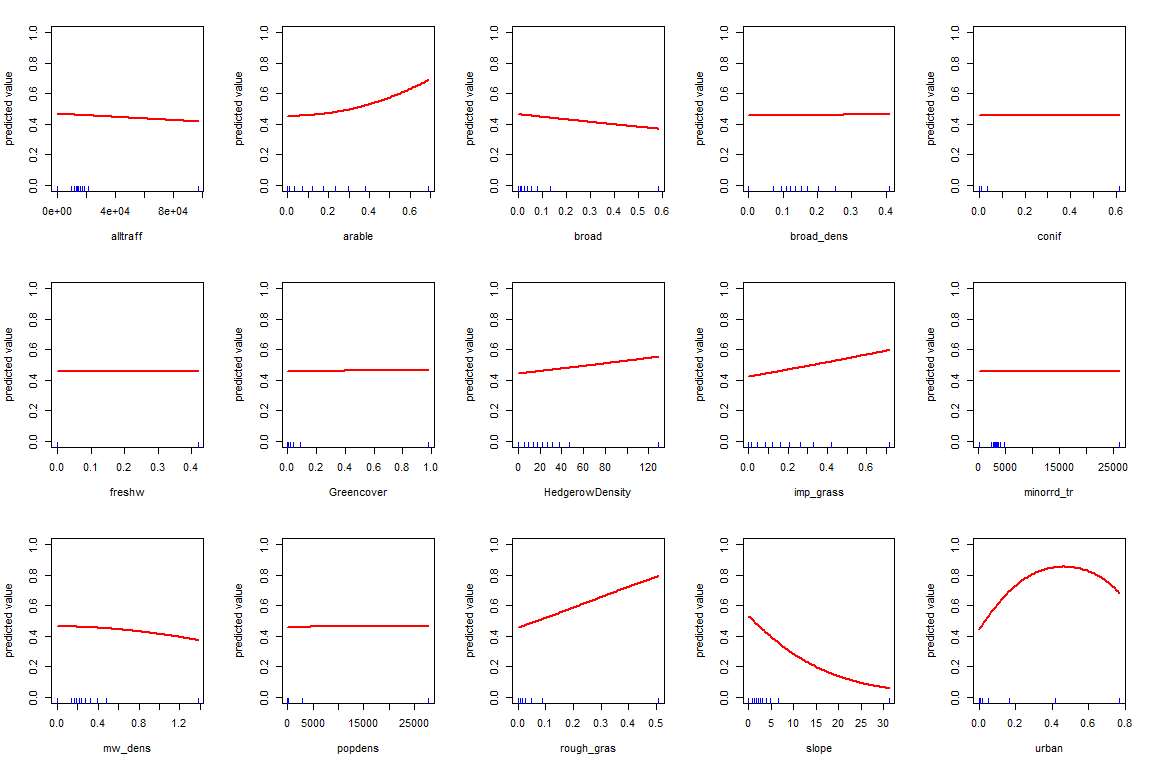


Supplementary 9: Summary of variable importance and response curves of the national model (roadkill background data).


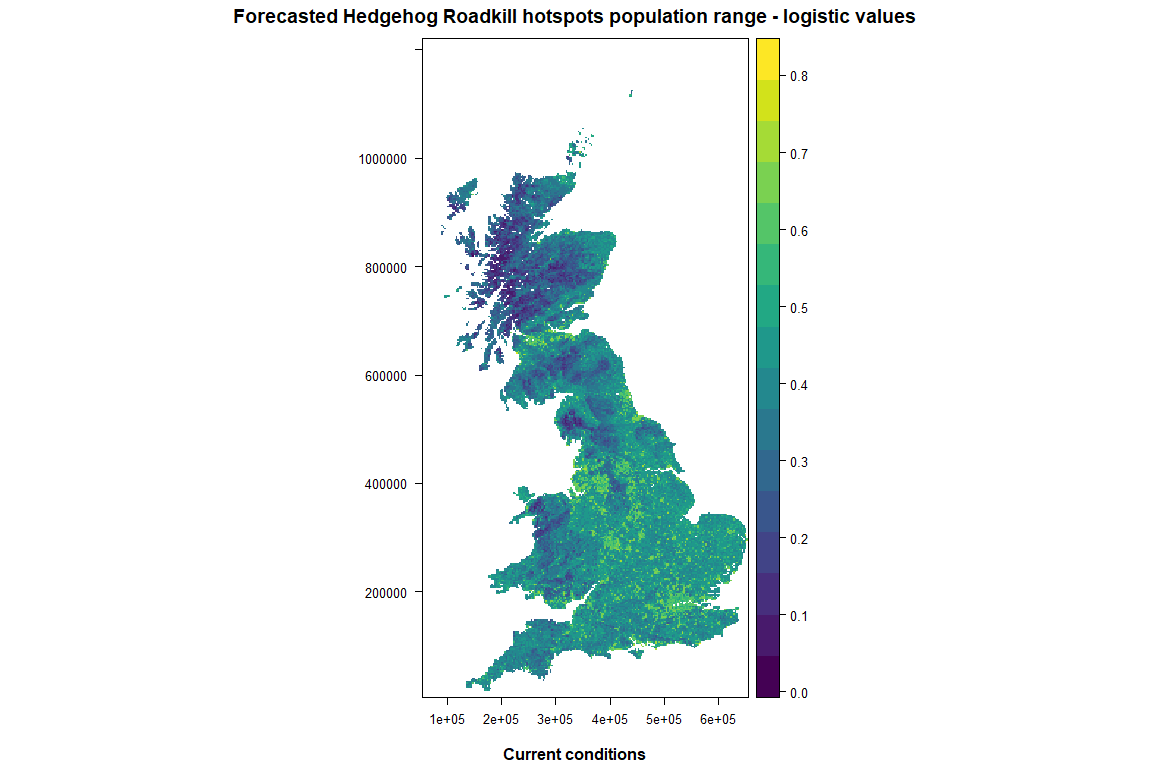


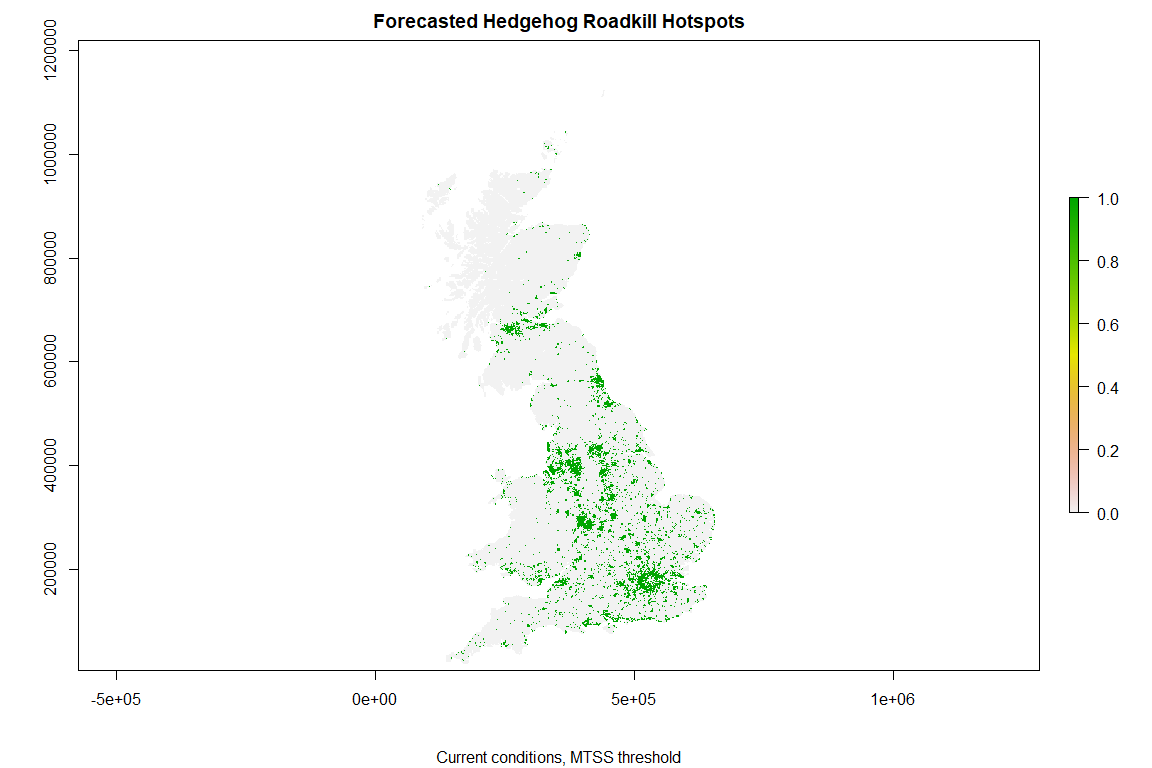


Supplementary 10: Mapped logistic habitat suitability indices (HSI) for hedgehog roadkill and forecasted high-risk of collision areas of the national model (roadkill background data).


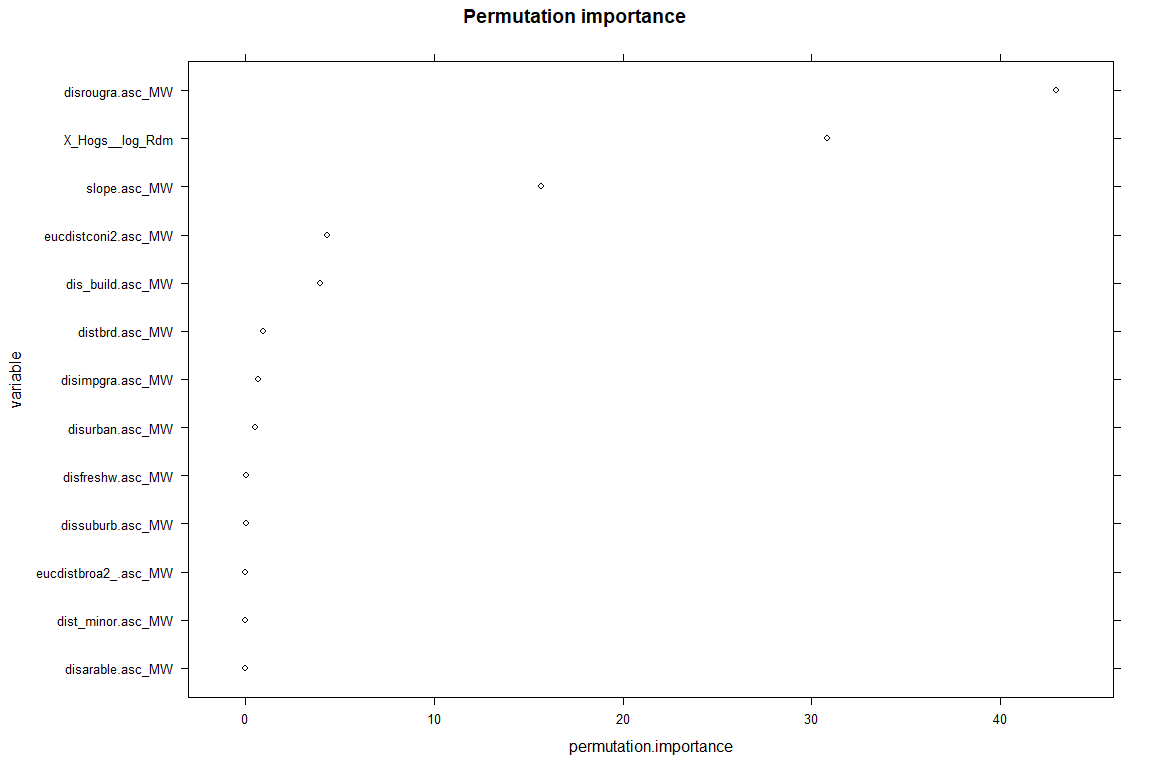


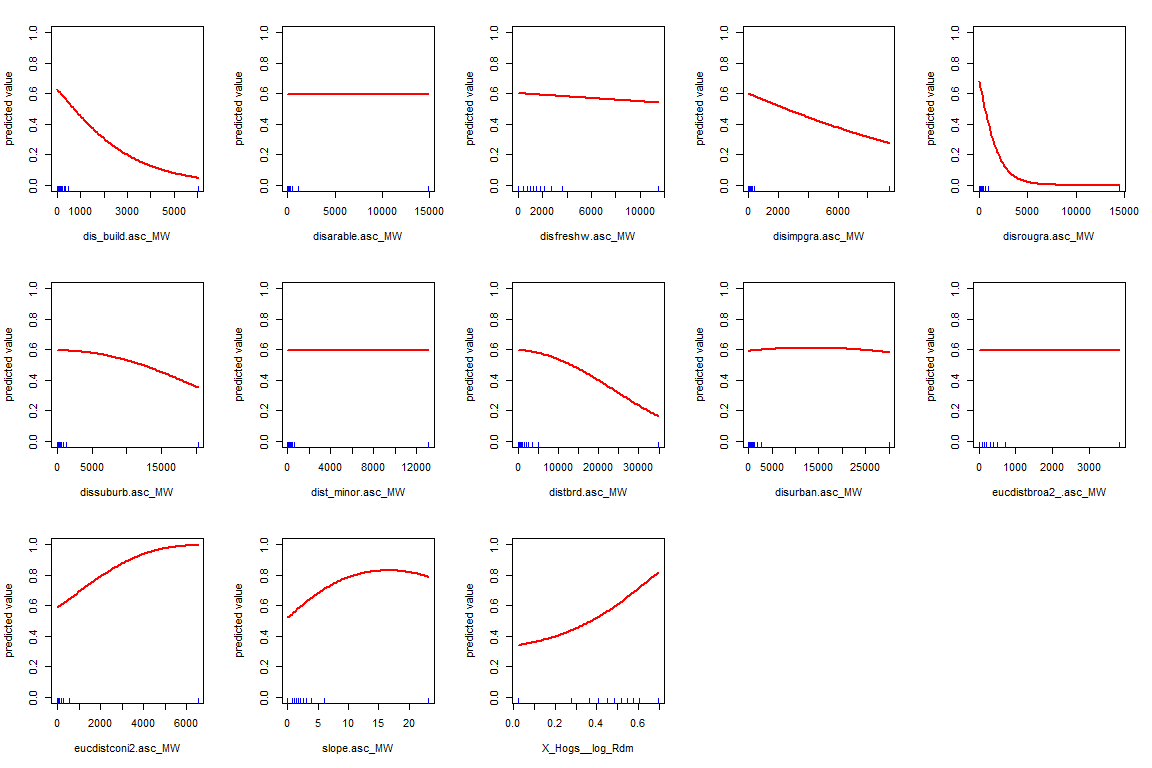


Supplementary 11: Summary of variable importance and response curves of the major road model (random background data)


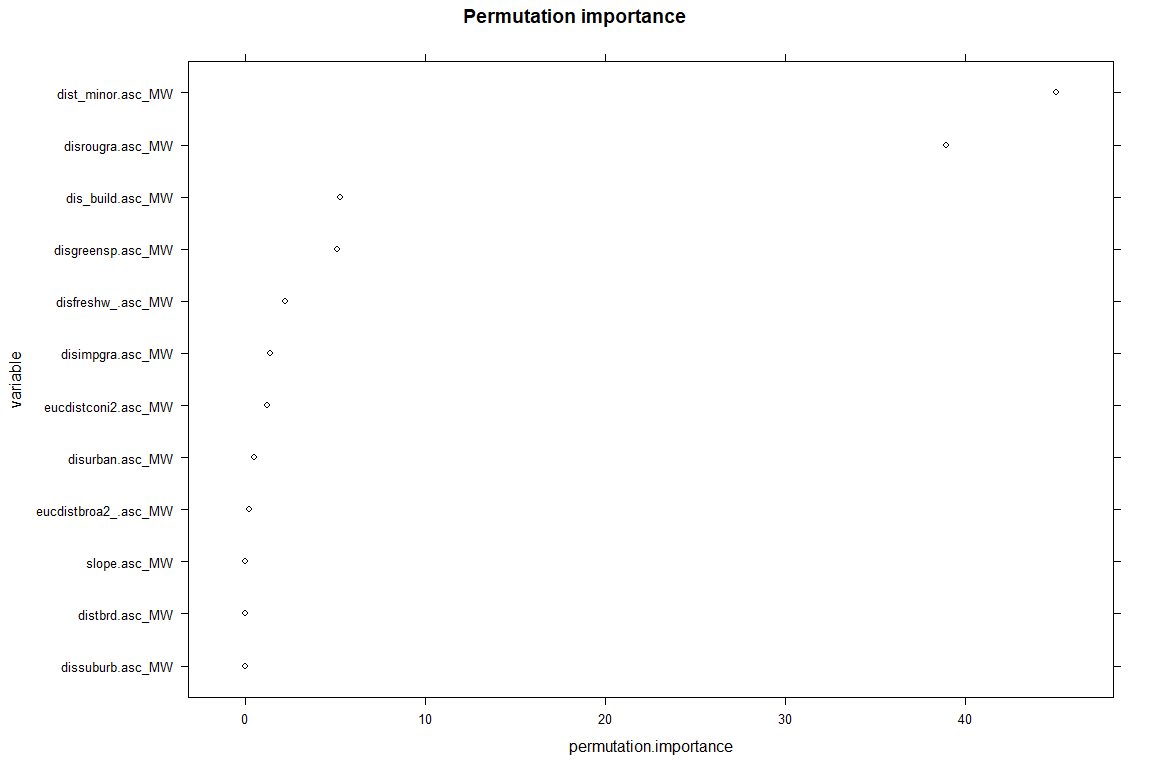


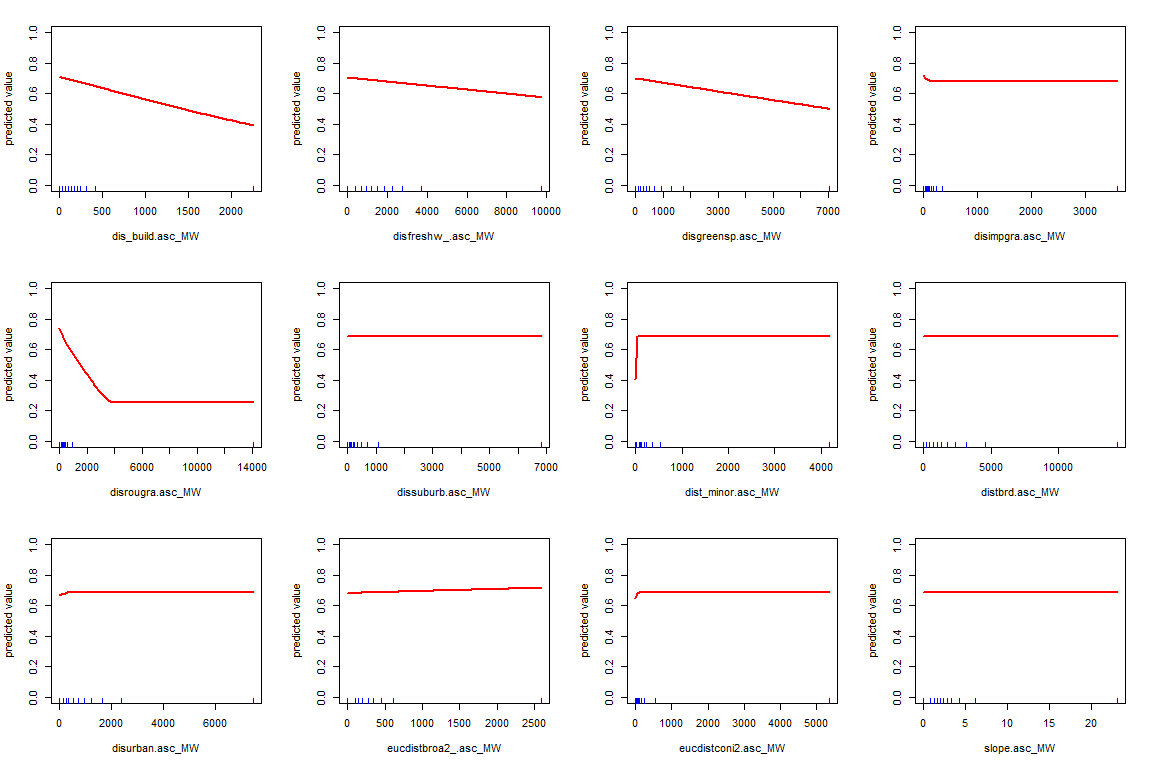


Supplementary 12: Summary of variable importance and response curves of the major road model (roadkill background data)


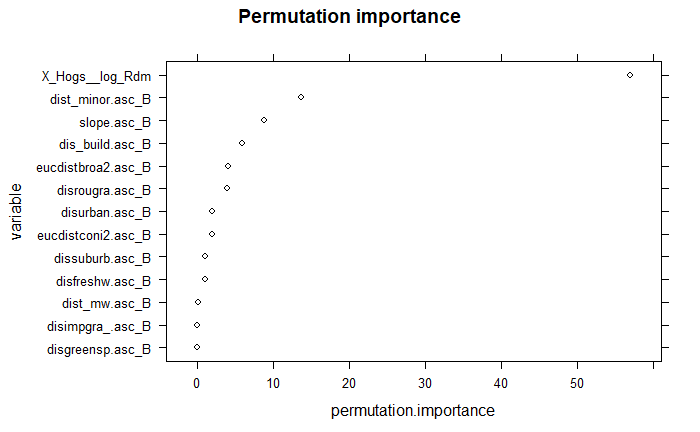


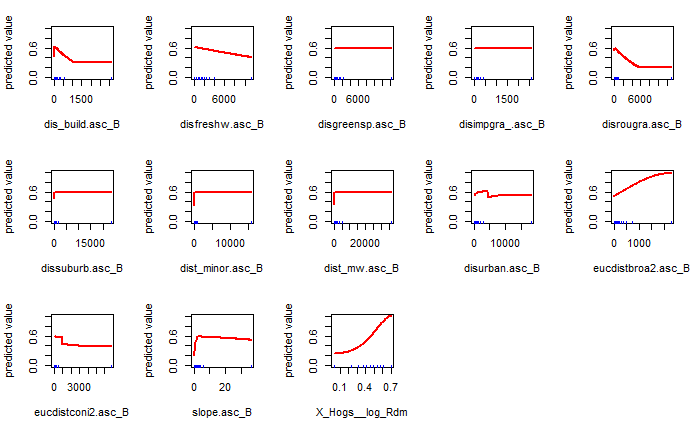


Supplementary 13: Summary of variable importance and response curves of the B road model (Random background data)


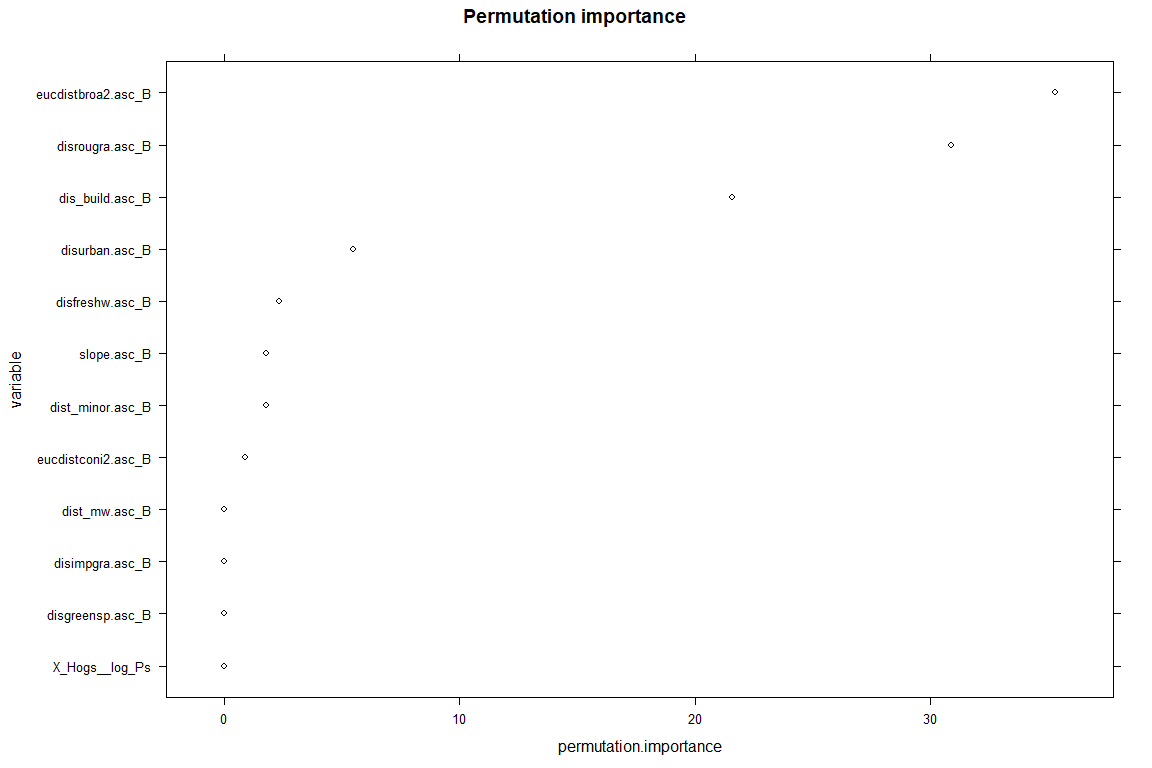


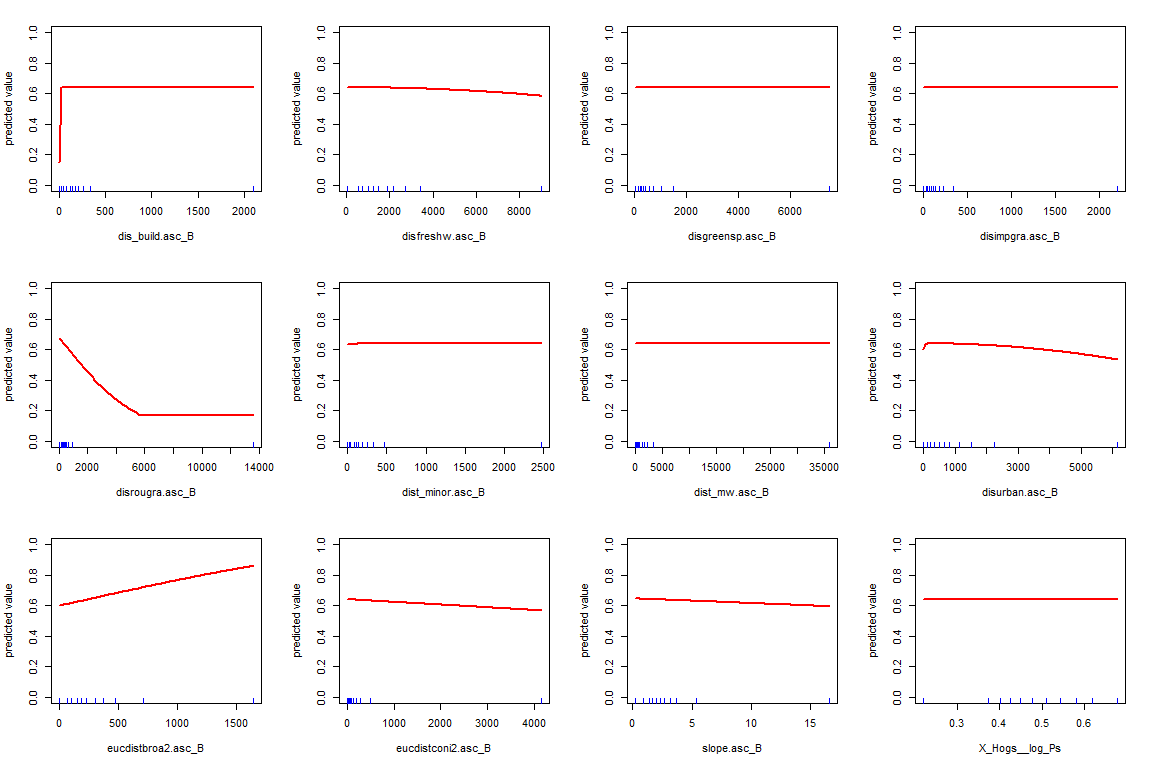


Supplementary 14: Summary of variable importance and response curves of the B road model (Roadkill background data)


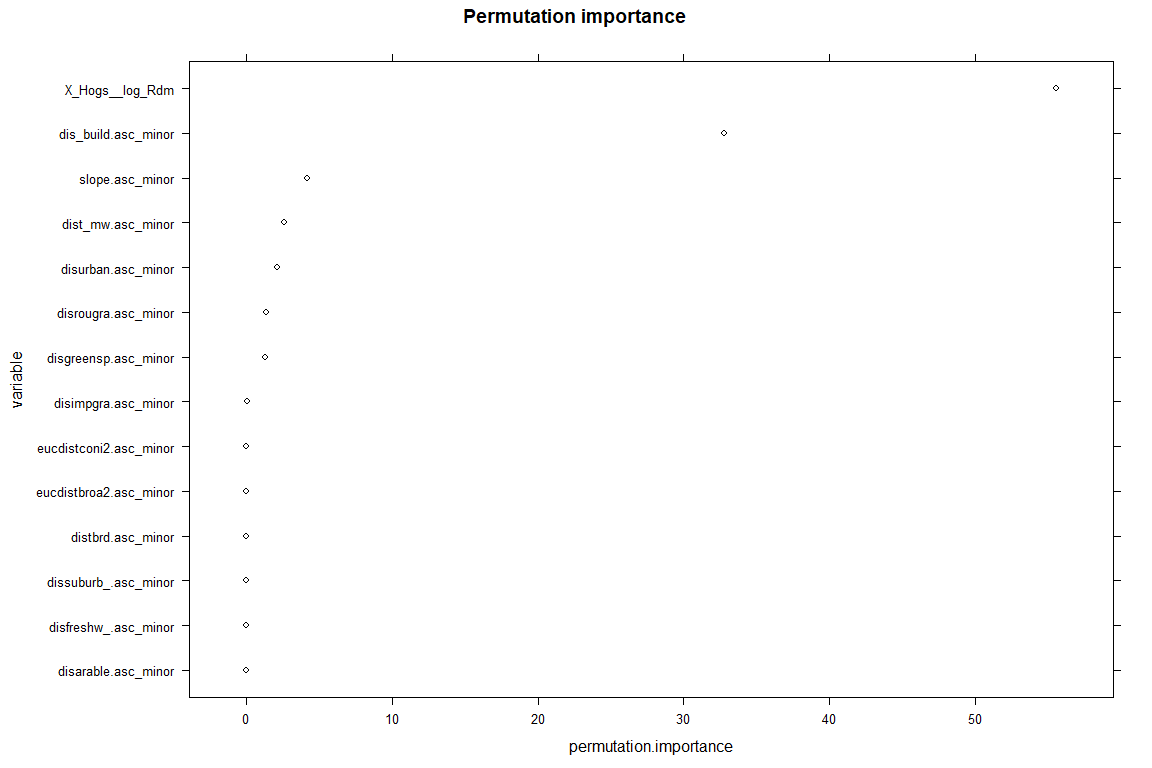


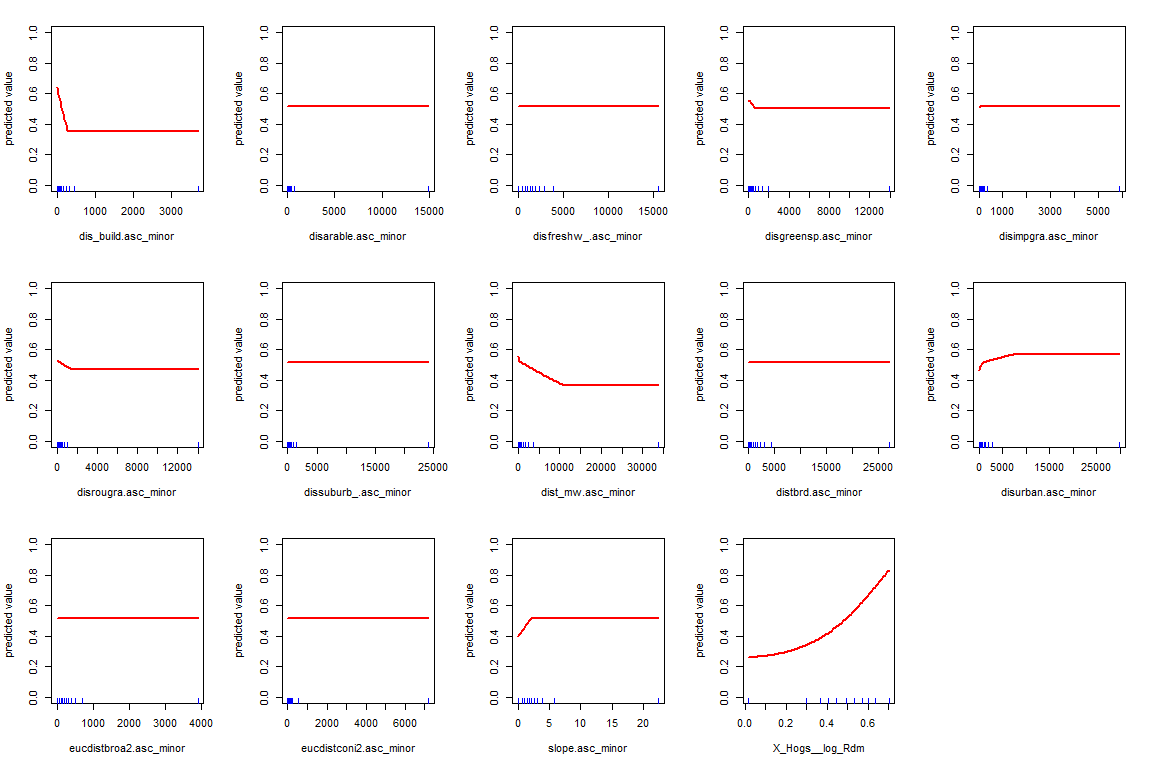


Supplementary 15: Summary of variable importance and response curves of the minor road model (Random background data)


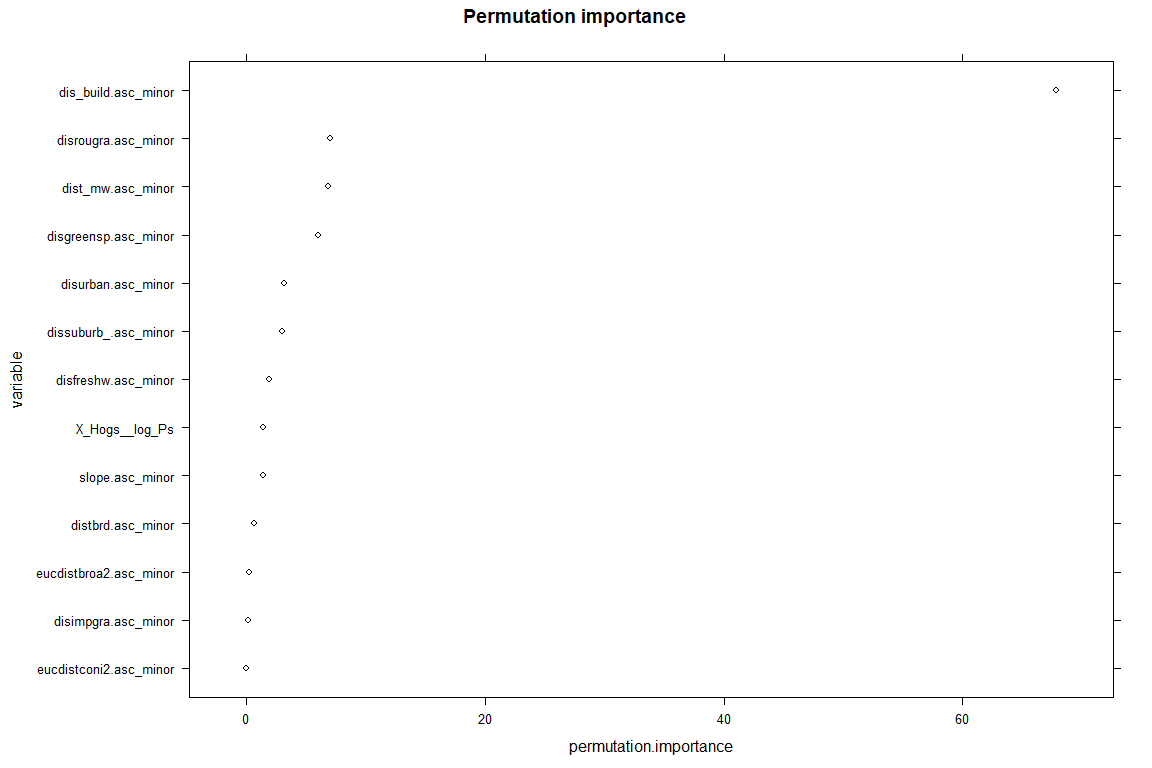


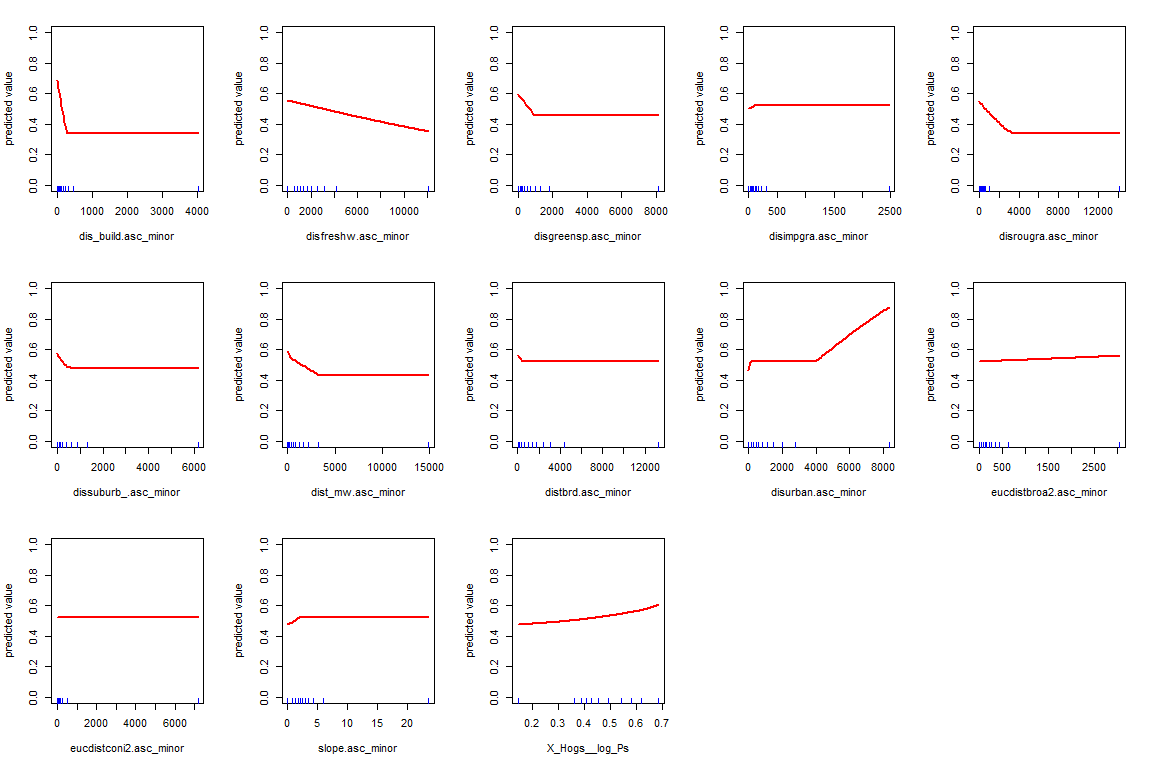


Supplementary 16: Summary of variable importance and response curves of the minor road model (Roadkill background data)


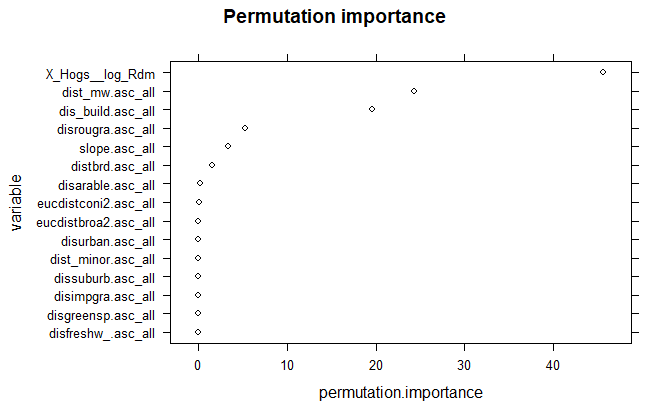


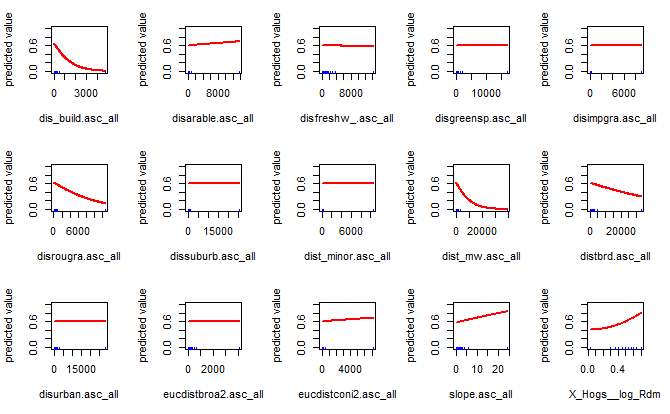


Supplementary 17: Summary of variable importance and response curves of the all roads model (Random background data)


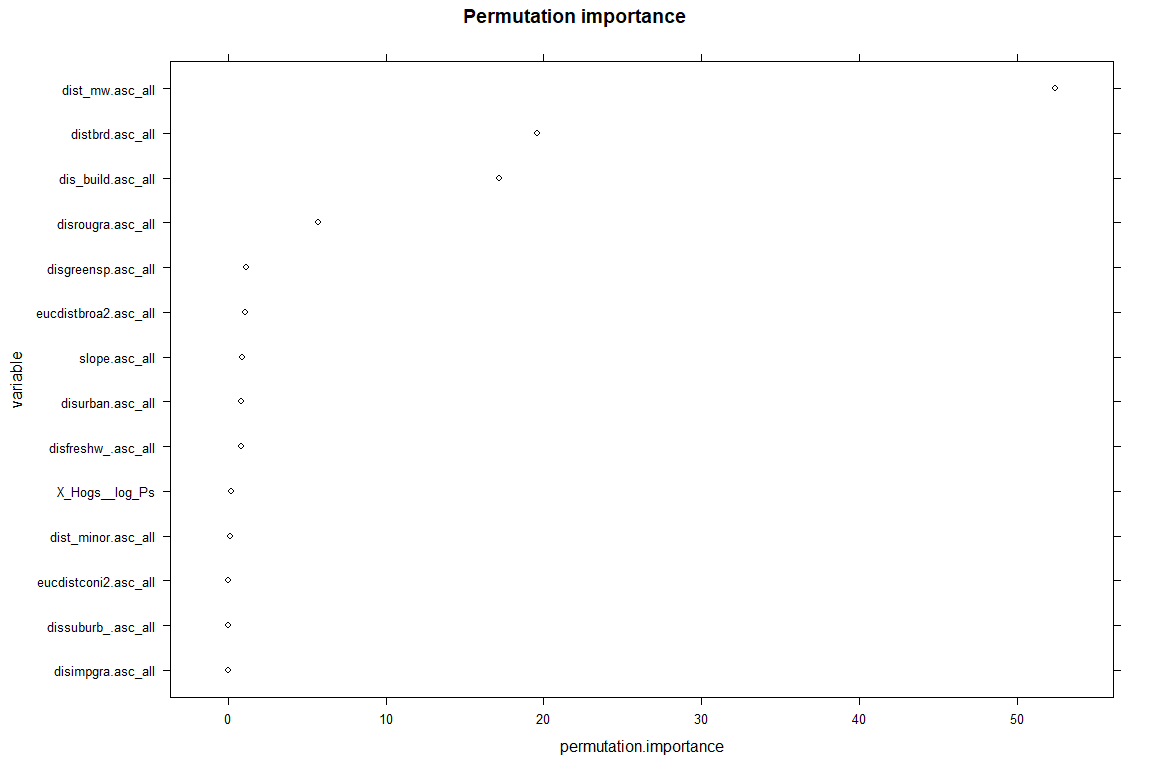


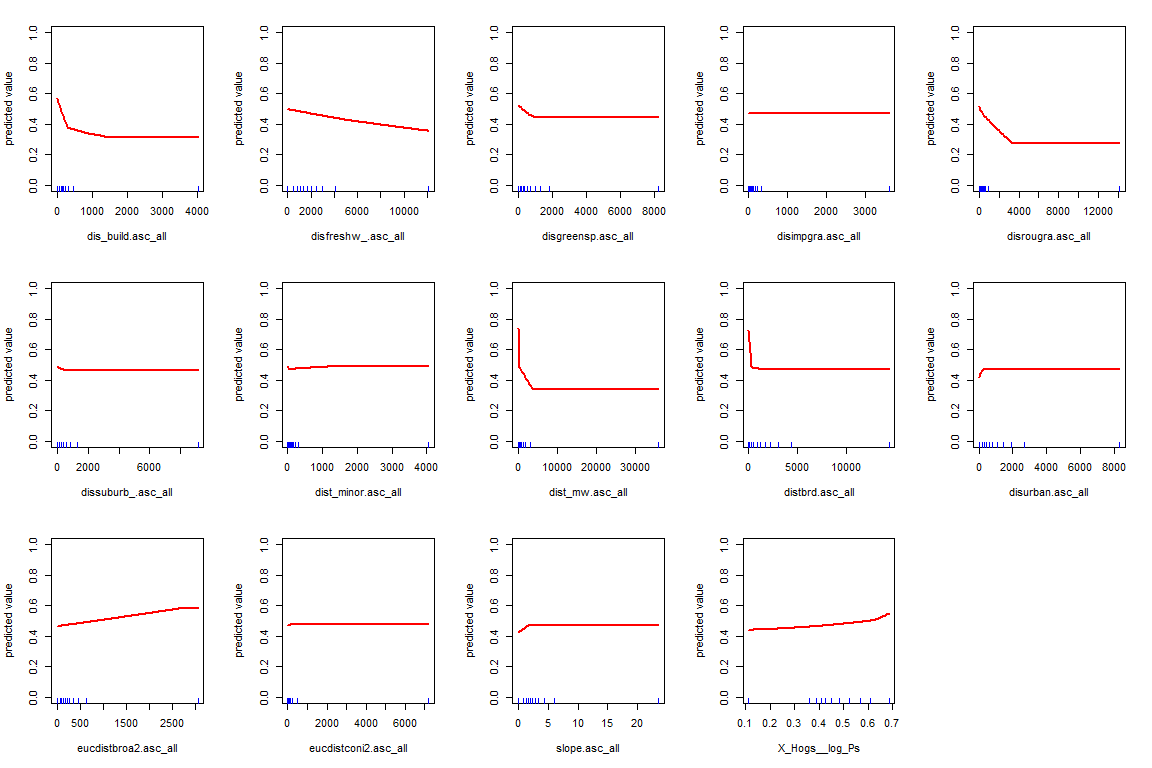


Supplementary 18: Summary of variable importance and response curves of the all roads model (Roadkill background data)


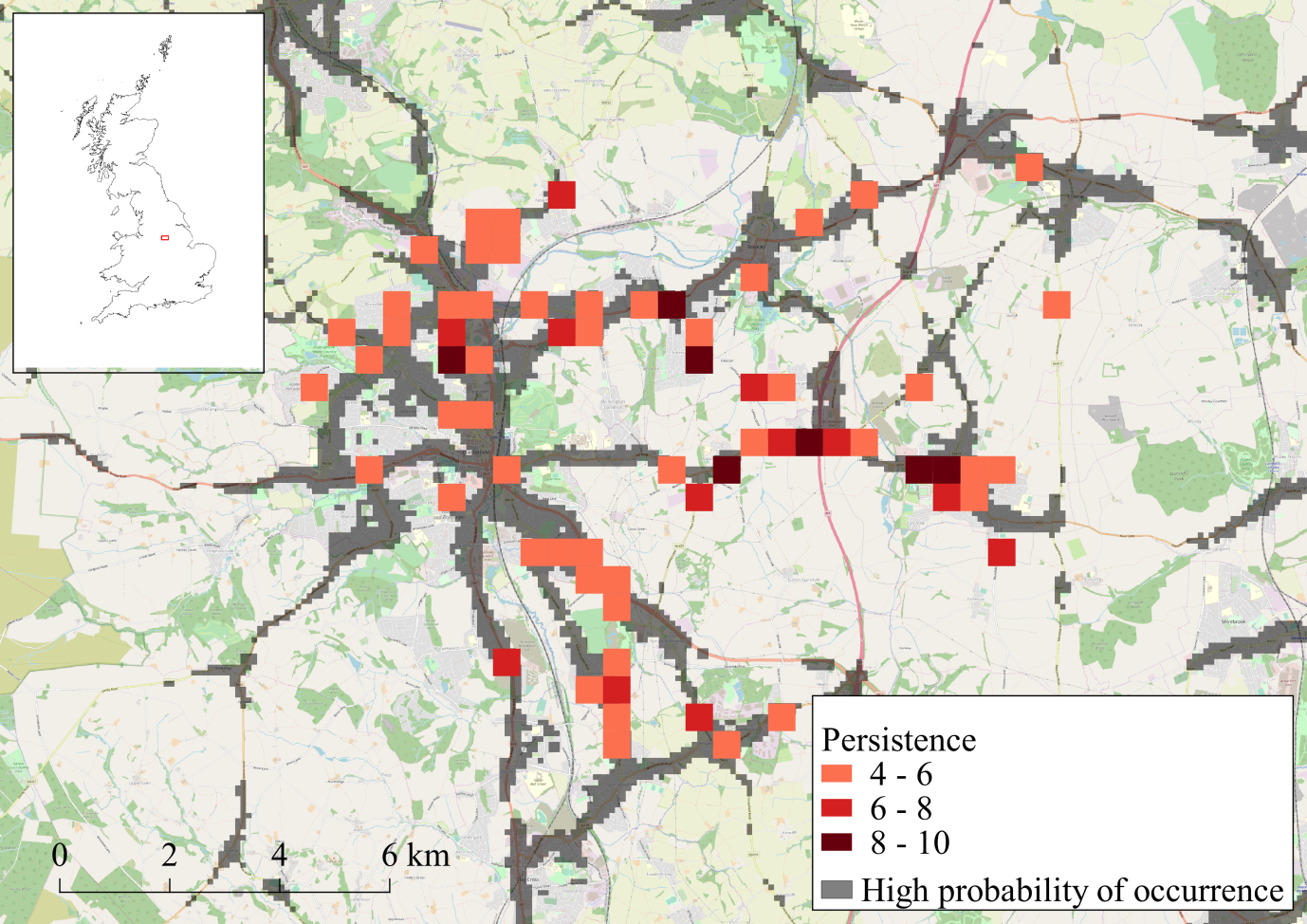


Supplementary 19: Using combined persistence data and predicted high-risk collision areas as a case study in Chesterfield to identify priority mitigation measures.
